# Id1 Represses Osteoclast-Dependent Transcription and Affects Bone Formation and Hematopoiesis

**DOI:** 10.1371/journal.pone.0007955

**Published:** 2009-11-24

**Authors:** April S. Chan, Kristian K. Jensen, Dimitris Skokos, Stephen Doty, Hannah K. Lederman, Rosandra N. Kaplan, Shahin Rafii, Stefano Rivella, David Lyden

**Affiliations:** 1 Department of Pediatrics and Department of Cell and Developmental Biology, Weill Cornell Medical College, New York, New York, United States of America; 2 Department of Pediatric Hematology-Oncology, Children's Cancer and Blood Foundation Laboratories, Weill Medical College of Cornell University, New York, New York, United States of America; 3 Laboratory of Molecular Immunology, The Rockefeller University, New York, New York, United States of America; 4 Hospital for Special Surgery, New York, New York, United States of America; 5 Memorial Sloan-Kettering Cancer Center, New York, New York, United States of America; 6 Department of Genetic Medicine, Weill Cornell Medical College, New York, New York, United States of America; KU Leuven, Belgium

## Abstract

**Background:**

The bone-bone marrow interface is an area of the bone marrow microenvironment in which both bone remodeling cells, osteoblasts and osteoclasts, and hematopoietic cells are anatomically juxtaposed. The close proximity of these cells naturally suggests that they interact with one another, but these interactions are just beginning to be characterized.

**Methodology/Principal Findings:**

An *Id1^−/−^* mouse model was used to assess the role of Id1 in the bone marrow microenvironment. Micro-computed tomography and fracture tests showed that *Id1^−/−^* mice have reduced bone mass and increased bone fragility, consistent with an osteoporotic phenotype. Osteoclastogenesis and pit formation assays revealed that loss of *Id1* increased osteoclast differentiation and resorption activity, both *in vivo* and *in vitro*, suggesting a cell autonomous role for Id1 as a negative regulator of osteoclast differentiation. Examination by flow cytometry of the hematopoietic compartment of Id1*^−/−^* mice showed an increase in myeloid differentiation. Additionally, we found increased expression of osteoclast genes, *TRAP*, *Oscar*, and *CTSK* in the *Id1^−/−^* bone marrow microenvironment. Lastly, transplantation of wild-type bone marrow into *Id1^−/−^* mice repressed *TRAP*, *Oscar*, and *CTSK* expression and activity and rescued the hematopoietic and bone phenotype in these mice.

**Conclusions/Significance:**

In conclusion, we demonstrate an osteoporotic phenotype in *Id1^−/−^* mice and a mechanism for Id1 transcriptional control of osteoclast-associated genes. Our results identify *Id1* as a principal player responsible for the dynamic cross-talk between bone and bone marrow hematopoietic cells.

## Introduction

Hematopoietic stem and progenitor cell (HSPC) self-renewal, proliferation, and differentiation are tightly regulated processes that depend on the microenvironment in which they reside [1], [2]. This microenvironment is located in the bone marrow (BM) where hematopoietic and bone remodeling cells, osteoblasts and osteoclasts, are anatomically juxtaposed. Due to the close proximity of these cells, it is reasonable to hypothesize that these hematopoietic and bone cells communicate with and/or regulate one another. However, the complex microenvironmental interactions and the molecular pathways that govern these connections occurring at this bone-bone marrow interface are still not well understood.

At the bone surface, the dynamic process of bone remodeling involves the constant interaction between bone-forming osteoblasts and bone-resorbing osteoclasts [3]–[7]. While the roles of osteoblasts and osteoclasts in bone remodeling have been well established, recent studies have demonstrated that these cells are also crucial components of the hematopoietic stem cell (HSC) niche. Accumulating evidence suggests that osteoblasts enforce the quiescence of HSCs, while osteoclasts induce hematopoietic activity. In particular, osteoblasts have been shown to physically anchor HSCs to the endosteal surface of the trabecular regions of the bone, thereby keeping them in an undifferentiated and quiescent state [8]–[10]. Conversely, osteoclasts secrete bone-resorbing enzymes, such as cathepsin K (CTSK), which promote mobilization of HSCs from their quiescent state. This process involves the cleavage of cytokines, such as stem cell factor (SCF), that are responsible for HSC anchorage to the niche [11], [12]. Thus, the balance between osteoblastic and osteoclastic activity not only dictates healthy bone volume but also contributes to maintaining hematopoietic homeostasis.

Although a great deal of progress has been made in defining the BM microenvironment, the molecular regulators involved, their interactions, and how they affect bone remodeling, hematopoiesis, and cell fate decisions are largely unknown. Here, we report a new role for the inhibitor of differentiation (*Id1*) gene in bone remodeling and maintenance of the BM microenvironment. Id1 is one of four proteins in a family of transcriptional regulators that all function by inhibiting basic helix-loop-helix (bHLH) transcription factors from binding DNA [13], [14]. Id proteins have a helix-loop-helix domain that allows them to dimerize with bHLH transcription factors, but lack a DNA-binding domain. As a result, Id-bHLH transcription factor heterodimers cannot bind to DNA, leading to the disruption of the events mediating the cellular differentiation and proliferation of a variety of different cell types.

In this study, we report several lines of evidence that Id1 is a critical regulator of bone and BM homeostasis. In particular, *Id1^−/−^* mice exhibited an osteoporotic phenotype, with significantly reduced bone mineral content and density. This is a novel finding, as Id1 has not been previously linked to osteoporosis. We also show that the expression of genes necessary for osteoclast maturation, such as *CTSK*, *TRAP*, and *Oscar* are upregulated in osteoclasts derived from *Id1^−/−^* mice and repressed in osteoclasts overexpressing *Id1*, suggesting that Id1 is a master regulator of a set of genes required for osteoclast function and bone resorption. Examination of the hematopoietic compartment of *Id1^−/−^* mice revealed an increase in myeloid differentiation and HSPC proliferation. Thus, in the absence of Id1, the HSPC pool is likely depleted by the increased flux towards myeloid differentiation, resulting in osteoclast-driven changes to the BM microenvironment. Results from this study will shed light on the molecular cues at the bone-bone marrow interface that regulate both bone and BM homeostasis. Moreover, Id1 may potentially be a novel therapeutic target for the treatment of skeletal disorders.

## Results

### *Id1^−/−^* Mice Exhibit an Osteoporotic Phenotype

**Figure 1:**
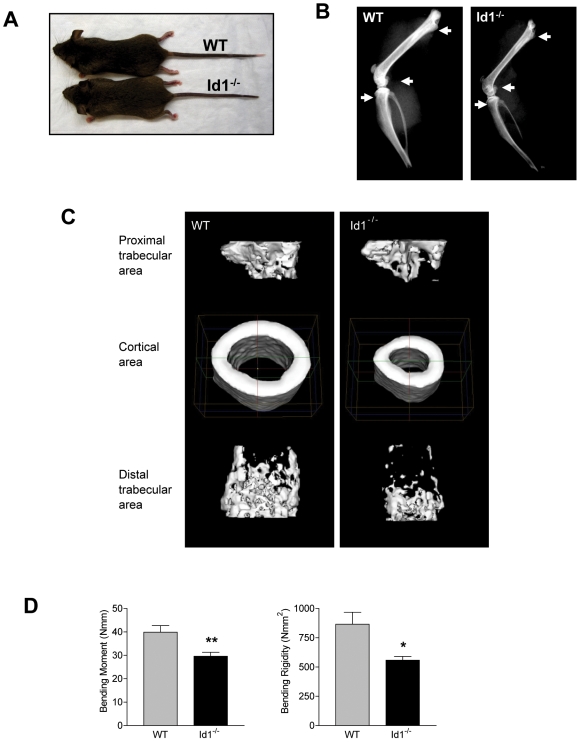
Skeletal phenotype of *Id1^−/−^* and wild-type mice at 6 weeks of age. (A) Photograph of a representative *Id1^−/−^* and wild-type mouse. (B) X-ray microradiographs of hindlimbs. White arrows indicate trabecular bone regions. (C) Representative micro-CT images of the femoral trabecular and cortical regions in wild-type and *Id1^−/−^* mice. (D) Three-point bending tests for evaluating the mechanical strength of femurs from wild-type (n = 8) and *Id1^−/−^* (n = 8) mice. The parameters measured include the bending moment and the bending rigidity (*P<0.05, **P<0.01). Error bars represent ±S.E.M.

To assess the effect of loss of Id1 on skeletal structure and function, we analyzed the bones of *Id1^−/−^* mice. *Id1^−/−^* mice were born at the expected mendelian frequency and showed no obvious abnormalities. We observed that *Id1^−/−^* mice were slightly, but significantly smaller than wild-type littermates (Figure 1A, Figure S1A). Morphological analysis of the skeleton revealed a phenotype that previously has not been reported in these mice. Microradiographs showed a marked decrease in the radiodensity of *Id1^−/−^* long bones, a feature that was most pronounced in regions of rapid longitudinal growth, such as the distal femur and the proximal tibia (Figure 1B). Three-dimensional microstructural analysis using micro-computed tomography (micro-CT) confirmed that loss of Id1 resulted in a significant reduction in bone mass (Figure 1C). Furthermore, this analysis revealed significant decreases in bone volume fraction, bone mineral content, bone mineral density (BMD), trabecular thickness, and marrow area (Table 1, Figure S1B). To determine whether the differences in the microstructural properties influenced the mechanical strength of the bone, we performed a three-point bending test on the femurs of wild-type and *Id1^−/−^* mice. The bending moment and bending rigidity were significantly decreased in *Id1^−/−^* mice (Figure 1D), indicating that the bones of *Id1^−/−^* mice are weaker and more prone to fractures as compared to wild-type bones. Together, these results demonstrate an osteoporotic phenotype in *Id1^−/−^* mice, characterized by low bone mass and micro-architectural deterioration of bone tissue, with a resultant increase in bone fragility and susceptibility to fracture.

**Table 1:** Characteristics of femurs in 6-week old wild-type and Id1^−/−^ mice.

	WT (n = 8)	Id1*^−/−^* (n = 8)	p-value
Bone Volume Fraction	0.199±0.024	0.135±0.021	<0.001
Bone Mineral Content (mg)	1.514±0.176	1.015±0.173	<0.001
Bone Mineral Density (mg/cc)	665.8±16.25	578.1±19.37	<0.01
Trabecular Thickness (mm)	0.035±0.003	0.031±0.003	<0.05
Marrow Area (mm2)	1.022±0.065	0.552±0.019	<0.001

### Osteoblast Development and Function Appear Normal in *Id1^−/−^* Mice

**Figure 2:**
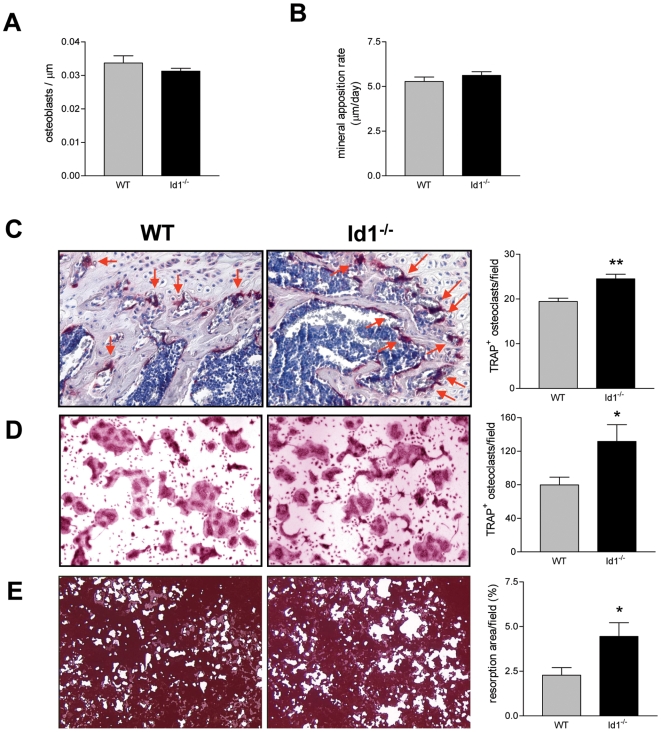
Enhanced osteoclast differentiation in *Id1^−/−^* mice. (A) Femoral sections were stained for Procollagen Type I and the number of osteoblasts per unit surface area was counted. (B) The mineral apposition rate was determined by double tetracycline labeling. (C) Enzyme histochemistry staining for TRAP on femoral sections (200X). Arrowheads indicate TRAP^+^ osteoclasts stained in red. (D) BM cells were cultured in the presence of M-CSF and RANKL for 3 days and then subjected to TRAP staining (400X). (E) BM cells were plated on a BD BioCoat Osteologic slide and cultured in the presence of M-CSF and RANKL for 10 days. The area resorbed was visualized by von Kossa staining (40X). (A–E) *P<0.05, **P<0.01; n = 5. Error bars represent ±S.E.M.

Since proper bone remodeling is determined by a delicate balance between the bone-forming osteoblasts and the bone-resorbing osteoclasts, the observed osteoporosis in *Id1^−/−^* mice could be a consequence of decreased osteoblast activity, increased osteoclast activity, or both. We first examined whether the loss of Id1 affects osteoblast development and function. Staining for osteoblast marker, pro-collagen I, on femur sections showed normal ratios of osteoblast numbers to bone surface in *Id1^−/−^* mice as compared to wild-type littermates (Figure 2A). Double tetracycline labeling also showed normal mineral apposition rates (Figure 2B), indicating normal osteoblast activity *in vivo*. In addition, the expression of osteoblast-associated cytokines receptor activator of nuclear factor-κB ligand (RANKL), osteoprotegerin (OPG), and stromal cell-derived factor 1 (SDF-1) was not altered in *Id1^−/−^* and wild-type bones (Figure S2). Thus, the decrease in bone mass in *Id1^−/−^* mice did not appear to be due to defects in osteoblast number or function.

### Id1 Negatively Regulates Osteoclastogenesis *In Vivo* and *InVitro*

Next, we examined whether Id1 affects osteoclast differentiation and function. Enzyme histochemistry for osteoclast marker, tartrate-resistant acid phosphatase (TRAP), on femur sections showed significantly increased osteoclast numbers in *Id1^−/−^* long bones (Figure 2C). To determine whether increased osteoclast differentiation could be caused by a cell-autonomous role for Id1in the osteoclast lineage, BM mononuclear cells were isolated and differentiated *in vitro*. In support of our *in vivo* findings, differentiation of *Id1^−/−^* BM cells gave rise to 1.6 fold more TRAP-positive osteoclasts as compared to wild-type cells (Figure 2D). Moreover, *in vitro* resorption assays showed that *Id1^−/−^* osteoclasts were functionally active (Figure 2E). Thus, our *in vivo* and *in vitro* data suggest that Id1 has a cell-intrinsic role as a negative regulator of osteoclast differentiation.

### Myeloid Differentiation Is Enhanced in *Id1^−/−^* Mice

**Figure 3:**
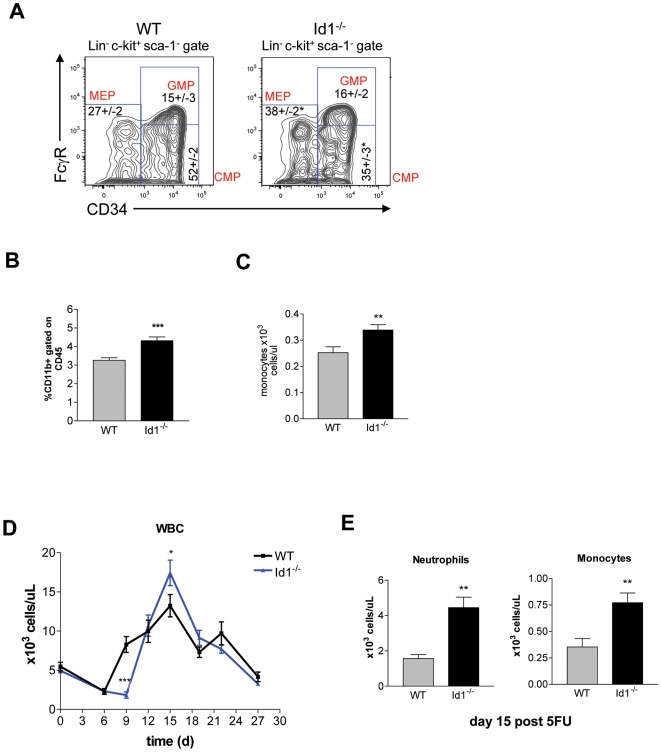
Enhanced myeloid differentiation in *Id1^−/−^* mice. (A) Representative flow-cytometric profiles of myeloid progenitor populations (CMP, GMP, MEP) in wild-type and *Id1^−/−^* BM (n = 6). (B) Monocytes in the BM were measured by flow cytometry using antibodies against CD11b and CD45 (n = 8). (C) Monocytes in the blood were measured by a complete blood count test (n = 40). (D) White blood cell (WBC) counts in wild-type and *Id1^−/−^* mice during hematopoietic challenge. Mice were given a single injection of 5-FU and bled every 3 days (n = 5). (E) Monocyte and neutrophil counts measured 15 days after 5-FU injection (n = 5). (A–E) *P<0.05, **P<0.01, ***P<0.001. Error bars represent ±S.E.M.

Whereas osteoblasts arise from mesenchymal stem cells, osteoclasts differentiate from hematopoietic monocyte/macrophage precursors of the myeloid lineage. Consequently, the observed osteoporotic bone phenotype and increased osteoclastogenesis in *Id1^−/−^* mice could be due to altered homeostasis of myeloid precursor cells. To explore this possibility, we analyzed various subsets of committed myeloid progenitor cells in the BM, represented by the lineage negative, c-kit^+^ and sca-1^−^, (LKS^−^) progenitor population. The common myeloid progenitor (CMP) population, the most immature of the myeloid progenitor cells, was decreased in *Id1^−/−^* mice, while the more mature megakaryo-erythrocyte progenitor (MEP) subset was significantly expanded (Figure 3A). Additionally, analysis of differentiated myeloid cell subpopulations showed a higher fraction of CD11b^+^ monocytes in the BM (Figure 3B) and a higher fraction of monocytes and neutrophils in the peripheral blood of *Id1^−/−^* mice compared to wild-type littermates (Figure 3C, Table S1). Thus, in Id1-deficient mice, myeloid differentiation is increased, indicating that Id1 normally acts to maintain myeloid progenitors in an undifferentiated state. These findings are consistent with the previously suggested role of Id1 in restraining both myeloid [15] and erythroid [16] differentiation.

To determine whether the loss of Id1 alters the kinetics of myeloid differentiation, we challenged *Id1^−/−^* and wild-type mice with a single, low dose of 5-fluorouracil (5-FU), a myeloablative drug that mobilizes stem cells from their quiescent state to reconstitute the hematopoietic system (Figure 3D). We observed an increase in total numbers of white blood cells at day 15 after 5-FU administration due to an increase in both neutrophils and monocytes (Figure 3E), demonstrating that Id1 also regulates myeloid differentiation during hematopoietic recovery after myeloablation.

### Proliferation of *Id1^−/−^* LSK Cells Is Enhanced *In Vivo*, but Not *In Vitro*

**Figure 4:**
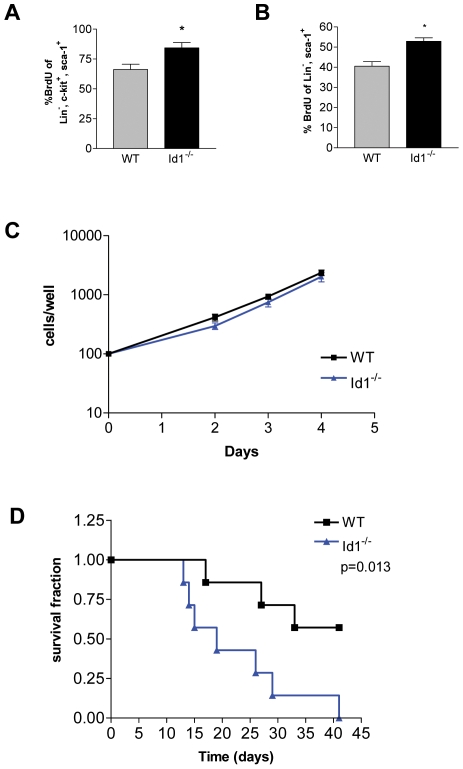
Enhanced proliferation of LSK cells in *Id1^−/−^* mice. (A) *In vivo* proliferation of LSK cells, at steady state, assessed by flow cytometry for the percentage of BrdU^+^ LSK cells (*P<0.05; n = 5). (B) *In vivo* proliferation of LSK cells from mice challenged with a single injection of 5-FU. Flow cytometry was performed 15 days post 5-FU to evaluate the percentage of BrdU^+^ LSK cells (*P<0.05; n = 7). (C) *In vitro* proliferation of sorted LSK cells in response to cytokine stimulation (n = 3). (D) Survival over time of wild-type and *Id1^−/−^* mice treated with weekly injections of 5-FU (P = 0.013; n = 10). Error bars represent ±S.E.M.

After a single, low dose injection of 5-FU, hematopoietic recovery in *Id1^−/−^* mice was delayed by 3 days, as compared to wild-type littermate controls (Figure 3D). To test whether this delay was due to increased cell cycling of *Id1^−/−^* progenitor cells, we performed BrdU labeling at steady state and during reconstitution following myeloablation. We observed a significant increase in the number of BrdU-labeled, Lin^−^ sca-1^+^c-kit^+^ (LSK) cells both at steady state (Figure 4A) and during hematopoietic recovery (Figure 4B), suggesting that a higher fraction of Id1 deficient LSK cells enter the cell cycle as compared to wild-type cells. In contrast, when we isolated and cultured the LSK cells *in vitro*, *Id1^−/−^* and wild-type cells proliferated at the same rate (Figure 4C), signifying that Id1 might indirectly affect the proliferation of LSK cells via factors secreted in the BM microenvironment.

To investigate whether the enhanced proliferation status of LSK cells in *Id1^−/−^* mice would lead to premature exhaustion of HSPCs after continuous myeloablative stress, we treated wild-type and *Id1^−/−^* mice weekly with 5-FU. We found that the survival rate was much lower in *Id1^−/−^* mice than in wild-type controls (Figure 4D). Analysis of blood and BM from both *Id1^−/−^* and wild-type mice severely affected by the 5-FU treatment showed a complete absence of myeloid cells, low hemoglobin/RBC counts, and low platelet counts (data not shown). These observations suggested that both *Id1^−/−^* and wild-type mice succumbed to BM failure, but that *Id1^−/−^* mice did so at a faster rate. Thus, Id1 deficient LSK cells showed altered proliferation kinetics with more progenitor cells entering cell cycle both at steady state and under conditions that lead to hematopoietic stress.

### Intrinsic and Extrinsic Mechanisms Operate in the Bone Marrow Microenvironment

**Figure 5:**
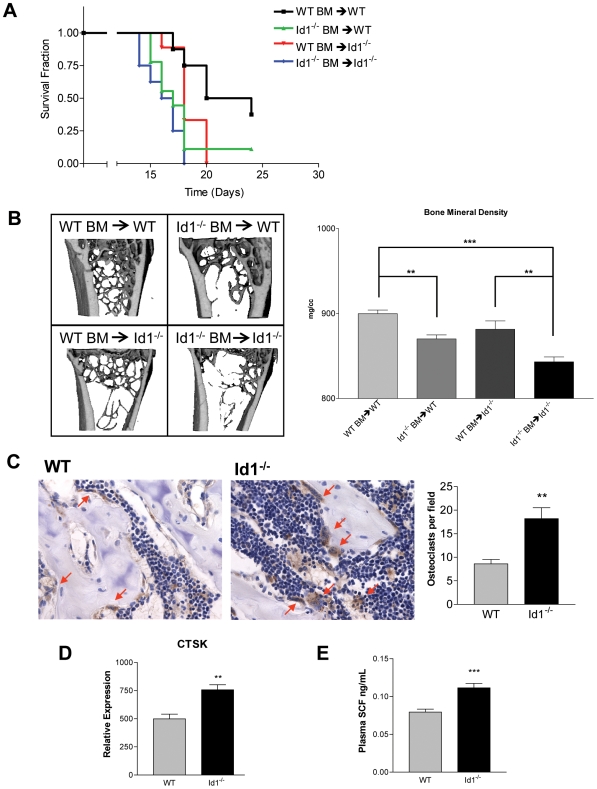
Loss of Id1 alters extrinsic niche factors. (A) Survival over time of BM transplanted mice treated with weekly injections of 5-FU. Lethally irradiated wild-type and *Id1^−/−^* mice were transplanted with either wild-type or *Id1^−/−^* BM. After 6 weeks to allow for hematopoietic reconstitution, the mice were injected weekly with 5-FU and their survival was monitored (n = 10). (B) Representative micro-CT images of the femoral distal trabecular region and bone mineral density measurements in BM transplanted mice (**P<0.01, ***P<0.001; n = 5). (C) CTSK staining of femoral sections (400X). Arrowheads indicate CTSK^+^ osteoclasts stained in brown (**P<0.01; n = 5). (D) Level of expression of CTSK in the BM of wild-type and *Id1^−/−^* mice (**P<0.01; n = 8). (E) SCF levels in plasma assayed by ELISA (***P<0.001; n = 8). Error bars represent ±S.E.M.

Recently, a number of studies have highlighted the importance of both intrinsic and extrinsic cues within the BM to maintain effective control over HSPCs [17], [18]. To explore the relative contributions of these intrinsic and extrinsic signals in the BM, we performed a series of BM transplant experiments in which we subjected the mice to weekly injections of 5-FU. Wild-type BM was transplanted into *Id1^−/−^* mice and *Id1^−/−^* BM was transplanted into wild-type mice. As a control, wild-type BM was transplanted into wild-type mice and *Id1^−/−^* BM was transplanted into *Id1^−/−^* mice. For the purpose of following engraftment by Y chromosome specific PCR, all recipient mice were female and all donor mice were male. Six weeks after transplantation, these mice were treated weekly with 5-FU and their survival was monitored (Figure 5A). If Id1 is exclusively an intrinsic regulator, we would expect a complete rescue of the hematopoietic phenotype, such that *Id1^−/−^* mice transplanted with wild-type BM would have the same survival rate as wild-type mice transplanted with wild-type BM. However, the hematopoietic rescue that we observed was partial. Transplantation of wild-type BM into *Id1^−/−^* mice significantly increased their survival, but not to the level of wild-type mice transplanted with wild-type BM. Similarly, transplantation of *Id1^−/−^* BM into wild-type mice significantly reduced their survival, but not to the same level as *Id1^−/−^* mice transplanted with *Id1^−/−^* BM.

To investigate whether BM transplantation could have a similar effect in rescuing the bone phenotype seen in *Id1^−/−^* mice, we performed the same BM transplants as described above and analyzed the bone properties by micro-CT. To allow sufficient time for bone turnover to occur, we waited 3.5 months post-transplantation before sacrifice. Micro-CT analysis showed that transplantation of wild-type BM into wild-type mice and *Id1^−/−^* BM into *Id1^−/−^* mice recapitulated the same bone phenotype seen in wild-type and *Id1^−/−^* mice, respectively. *Id1^−/−^* mice transplanted with *Id1^−/−^* BM had significantly reduced BMD and trabecular bone compared with wild-type mice transplanted with wild-type BM (Figure 5B). Following the same reasoning as above, we would expect that if Id1 is exclusively an intrinsic regulator, we would see a complete rescue of the bone phenotype, such that *Id1^−/−^* mice transplanted with wild-type BM would have the same bone microstructural properties as wild-type mice transplanted with wild-type BM. However, once again, the rescue that we observed was partial. Transplantation of wild-type BM into *Id1^−/−^* mice significantly increased their BMD and trabecular bone, but not to the level of wild-type mice transplanted with wild-type BM. Correspondingly, transplantation of *Id1^−/−^* BM into wild-type mice significantly reduced their BMD and trabecular bone, but not to the same level as *Id1^−/−^* mice transplanted with *Id1^−/−^* BM. Together, these results show that the loss of Id1 impacts both intrinsic and extrinsic signals in the BM microenvironment.

To rule out the possibility that the partial rescue of hematopoietic and bone phenotypes in these mice was not due to post transplant donor/host chimerism, we performed qPCR for the Y chromosome specific, sex determining region (SRY) gene. Complete donor chimerism is often defined as the detection of greater than 95% donor DNA [19]. By this definition, all of the mice we transplanted achieved complete donor hematopoietic chimerism (Figure S3), indicating the sustained engraftment of donor-derived cells. Therefore, it is unlikely that autologous hematopoiesis had any impact on the partial rescue of phenotypes in these mice.

Next, we examined the microenvironment of *Id1^−/−^* and wild-type mice to explore differences that might be responsible for the observed phenotypes. Recently, it was shown that CTSK, a proteolytic enzyme secreted by osteoclasts, can cleave cytokines that regulate stem cell proliferation, survival, and mobilization [11]. We found that CTSK, was upregulated at the mRNA and protein level in the BM of *Id1^−/−^* mice as compared to wild-type littermates (Figure 5C,D). Expression levels of other cathepsins remained unchanged (Figure S4). One of the cytokines that can be cleaved by CTSK is SCF, which is involved in anchoring stem cells to the niche. Accordingly, we found elevated levels of SCF in the plasma (Figure 5E), likely due to increased processing of SCF by CTSK in the BM.

### A Mechanism for Id1 Function in Bone and the Bone Marrow Microenvironment

Our findings suggested that Id1 is critical for proper function of the BM microenvironment and that loss of Id1 impacts HSPC homeostasis via osteoclast-induced modifications in the bone. Thus, we hypothesized that Id1 acts to inhibit differentiation of both myeloid cells and osteoclasts, thereby keeping HSPCs quiescent and in the BM (Figure S5). In support of this model, we observed that osteoclast differentiation was increased in the absence of Id1, leading to increased secretion of CTSK. As a result, SCF cleavage by CTSK released soluble SCF into the circulation and mobilized HSPCs out of the BM.

**Figure 6:**
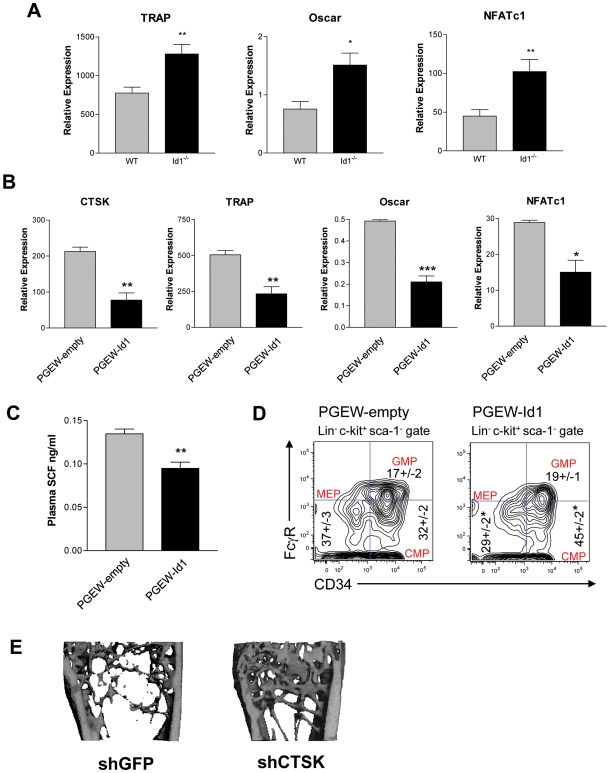
A proposed mechanism for Id1 function in the BM microenvironment. (A) Expression of osteoclast-associated genes, *TRAP*, *Oscar*, and *NFATc1* in the BM of wild-type and *Id1^−/−^* mice (n = 6). (B) Expression of osteoclast-associated genes from the BM of transplanted mice (n = 6). Lin^−^ BM cells from *Id1^−/−^* mice were transduced with lentivirus containing PGEW-Id1 or PGEW-empty vector overnight and transplanted into *Id1^−/−^* mice. After 8 weeks, the mice were sacrificed and BM from the femur was collected for qPCR analysis. (C) SCF levels in plasma assayed by ELISA. (D) Representative flow-cytometric profiles of myeloid progenitor populations (CMP, GMP, MEP) in mice transplanted with PGEW-Id1 or PGEW-empty BM (n = 6). (E) Representative micro-CT images of the femoral distal trabecular region in *Id1^−/−^* mice transplanted with BM containing a shRNA targeted against CTSK or GFP. (A-D) *P<0.05, **P<0.01, ***P<0.001. Error bars represent ±S.E.M.

Since Id proteins are known to negatively regulate bHLH transcription factor function, we hypothesized that Id1 interacts with specific bHLH transcription factors to inhibit osteoclast differentiation. Association between Id proteins and Mitf, a bHLH transcription factor that targets genes such as CTSK, TRAP, and osteoclast associated receptor (Oscar), has been demonstrated *in vitro* [20]. During osteoclast differentiation, Mitf has been shown to cooperate with other transcription factors, PU.1 and nuclear factor of activated T cells 1 (NFATc1), to synergistically induce expression of CTSK, TRAP, and Oscar [21]–[24]. In addition, a positive feedback loop has been described between Oscar and NFATc1, such that increased Oscar expression leads to increased NFATc1 expression [25]. To investigate the role of Id1 in transcriptional regulation of these osteoclast-associated genes, we performed qPCR for CTSK, TRAP, Oscar, and NFATc1 in BM isolated from *Id1^−/−^* and wild-type mice. In the absence of Id1, these genes were significantly upregulated (Figure 6A), suggesting that the loss of Id1 may allow increased binding of Mitf to PU.1 and NFATc1, subsequently resulting in increased expression of target genes.

To explore this further, we transplanted lethally irradiated *Id1^−/−^* mice with *Id1^−/−^* BM that was transduced with a lentivirus vector overexpressing *Id1* (Figure S6A, B). In these transplanted mice, the genetic expression profile was reversed. *CTSK*, *TRAP*, *Oscar*, and *NFATc1* expression were significantly downregulated in mice overexpressing *Id1*, as compared to mice receiving the empty vector control (Figure 6B). Furthermore, overexpression of *Id1* in the BM of transplanted mice led to decreased plasma levels of SCF (Figure 6C) and the restoration of BM myeloid progenitor cell populations (Figure 6D).

Finally, we assessed whether phenotypic effects due to the loss of Id1 could be reversed by abrogating CTSK activity. We transplanted lethally irradiated *Id1^−/−^* mice with *Id1^−/−^* BM that was transduced with a lentivirus vector containing a shRNA targeted against CTSK (shCTSK) (Figure S7A). A shRNA targeted against GFP (shGFP) was used as a control. To allow sufficient time for bone turnover to occur, we performed micro-CT on the bones of these mice 3.5 months after transplantation. Mice transplanted with BM containing shCTSK had increased bone volume fraction, trabecular number and thickness (Figure 6E, Table S2, Figure S7B). In comparison, control mice exhibited a severely reduced and more separated trabecular bone network, similar to that of *Id1^−/−^* mice. Hence, inhibition of CTSK activity was able to restore trabecular bone in *Id1^−/−^* mice. Overall, our results support a model wherein Id1 interacts with bHLH transcription factor Mitf to inhibit transcriptional activation of osteoclast-associated genes, subsequently leading to regulation of bone remodeling and the BM microenvironment.

## Discussion

The roles for Id1 in differentiation, cell growth, senescence, angiogenesis, and tumorigenesis have been extensively studied [14], [26]–[29]. Yet, how Id1 deficiency contributes to aging-associated phenotypes, such as osteoporosis, has not been well characterized. Previous studies have indicated that Id proteins likely inhibit osteoclast differentiation from BM monocyte cells grown *in vitro* [20]. However, the present study is the first to demonstrate the direct correlation between Id1 and bone growth in an *in vivo* animal model. Several novel findings have been generated from this study. First, *Id1^−/−^* mice exhibited osteoporosis due to increased osteoclast differentiation, a phenotype that has not been previously described in these mice. Second, the osteoporotic phenotype was associated with a reduction in the number of reserve HSPCs and an increased tendency toward myeloid differentiation. Id1 impact on myeloid and osteoclast cell numbers and their shared microenvironment can help explain the functional significance of the drive toward the myeloid compartment, as seen in our results, as well as others [15], [30]. Thus, the *Id1^−/−^* mouse represents a model that simulates the bone and BM features seen in an aged host. Disruption of normal osteoclast function via the loss of Id1 had profound effects on the behavior and activity of cells in the BM microenvironment. These observations demonstrate that bone cells provide critical regulatory cues to the hematopoietic system, confirming that cross-talk between these different cell types at the bone-bone marrow interface does indeed occur.

The BM microenvironment is a specialized region of the bone responsible for a host of homeostatic functions, including maintenance of stem cell number, protecting stem and progenitor cells from exhaustion over the lifetime of an organism, and providing cues that dictate the self-renewal or differentiation of HSPCs to other cell types [1], [31]. Within this niche, the endosteum lines the inner surface of the bone and serves as the interface between the bone and BM. Even though traditionally, bone and BM functions have been regarded as distinct, unrelated processes, it is becoming increasingly clear that the bone remodeling cells, osteoblasts and osteoclasts, are crucial components of the BM microenvironment. Osteoblasts and osteoclasts interact with each other through cytokines secreted by either cell type, as well as by membrane-bound ligands and receptors to initiate intracellular signaling [3], [6]. Osteoblasts and BM stromal cells secrete two cytokines, macrophage colony stimulating factor (M-CSF) and RANKL, which are both necessary and sufficient to induce osteoclast differentiation. M-CSF induces the expression of RANK in osteoclast precursor cells, priming them to differentiate in response to RANKL [6]. In addition, osteoclasts stimulated with M-CSF undergo cytoskeletal reorganization, cell spreading and migration [32], [33]. The downstream effects of RANKL-induced signaling include the expression of *CTSK* and *TRAP*, genes which encode crucial bone-resorbing enzymes that are secreted into the BM microenvironment and stimulate mobilization of HSPCs [6], [11], [12], [34]. RANKL also induces expression of integrin α_v_β_3_, which plays an important role in osteoclast adhesion, migration, and cell signaling [33], [34]. Therefore, intrinsic and extrinsic regulatory pathways converge to maintain bone and BM homeostasis.

Despite recent studies that have recognized the importance of osteoblasts in regulating hematopoiesis [8], [9], [35], we did not find any differences in osteoblast number or function between *Id1^−/−^* mice and wild-type controls. However, the present study does show the importance of Id1 during osteoclast differentiation. Osteoclast numbers were increased in the bones of *Id1^−/−^* mice and differentiation of BM monocytes to osteoclasts was increased by Id1 deficiency, suggesting a cell-autonomous role for Id1 in osteoclastogenesis. In addition, the absence of Id1 resulted in increased expression of *CTSK*, *TRAP*, *Oscar*, and *NFATc1*, while overexpression of *Id1* decreased expression of these genes. Therefore, interaction between Id1 and Mitf may normally act to inhibit a cascade of gene expression events required for osteoclast differentiation.

As a consequence of the increased osteoclast differentiation and activity, we observed increased secretion of extrinsic factors, such as CTSK, into the BM microenvironment. In keeping with a previous report showing the potential of CTSK to cleave stem cell regulating cytokine SCF [11], we found increased plasma levels of SCF in *Id1^−/−^* mice. We propose that this alteration in the level of secreted factors in the microenvironment directly contributed to the increased tendency of *Id1^−/−^* LSK cells to enter the cell cycle, leading to increased turnover and premature exhaustion of HSPCs. *Id1^−/−^* mice reconstituted with BM overexpressing *Id1* were able to reestablish the levels of HSPCs and myeloid cells comparable to those observed in wild-type mice. Furthermore, knockdown of CTSK in the BM of *Id1^−/−^* mice decreased osteoclast function and enhanced trabecular bone formation. Finally, our finding that transplantation of wild-type BM into *Id1^−/−^* mice only partially restored the bone and hematopoietic phenotype in these animals provides more evidence that the intrinsic program of cells in the microenvironment integrate with multiple extrinsic molecules and pathways needed to regulate bone and BM homeostasis. Although the present data are highly suggestive that Id1 can play a regulatory role in both intrinsic and extrinsic cues necessary for maintaining a balanced microenvironment, the challenge for future investigations will be to sort out cell intrinsic versus cell extrinsic activities *in vivo*. Undoubtedly this will be a difficult task, as they are most certainly interconnected, but by doing so it will elucidate the precise molecular mechanisms responsible for the role of Id1 in the bone and BM.

Finally, it has been shown that Id proteins often have overlapping functions and can compensate for each other. Specifically, *Id1* and *Id3* were shown to have very similar expression patterns and targeting of both genes is often required to produce a phenotype [36]. Previously, we reported that tumor growth was inhibited more effectively in *Id1^+/−^*, *Id3^−/−^* than in *Id1^+/−^*, *Id3^+/+^* mice [29]. Similarly, other studies have shown that simultaneous targeting of *Id1* and *Id3*, compared to targeting of each gene alone, is more effective at inhibiting tumor growth and metastatic potential of breast, colorectal, gastric, and pancreatic cancers [37]–[40]. In the current study, the absence of *Id1* alone produced a significant bone and hematopoietic phenotype, suggesting that *Id3* does not compensate for *Id1* in this instance. However, we found that *Id3* expression is slightly elevated in the BM of *Id1^−/−^* mice compared to wild-type littermates (data not shown). The significance of this finding is so far unclear, but it will be of interest to observe if *Id1^−/−^*, *Id3^+/−^* mice have a more severe bone and hematopoietic phenotype compared to *Id1^−/−^*, *Id3^+/+^* mice. Clearly, more work will be necessary to elucidate the precise role of *Id3* in the bone and hematopoietic phenotypes described here.

It is clear that our understanding of the fundamental mechanisms underlying the connections between the bone and BM is still in its infancy. In the future, many fruitful discoveries may come from investigating the commonalities between these two systems. This study offers insight into the cellular and molecular interactions of Id1 in regulation of bone and BM physiology. Although the clinical relevance of our findings has yet to be determined, the knowledge acquired from our results has the possibility of translating into potential therapeutic treatments in the areas of osteoporotic and malignant diseases. Moreover, microenvironments have garnered increased attention in recent years, due to findings that highlight the importance of BM cells, stem cells, and niches in areas of diseases such as cancer. We previously showed the role of Id proteins in supporting the growth of primary tumors and metastatic lesions [29], [41]. We also demonstrated the importance of niches in metastatic spread of tumor cells [42]. From the results of the present study, it is intriguing to speculate that long-standing osteoporosis may result in a reduced HSC pool, thus impairing the mobilization of HSCs to future peripheral sites of metastasis. It would also be of interest to investigate whether cooperation between osteoclasts and HSPCs can create a favorable environment for tumor cells to engraft and proliferate in the bones. A better understanding of the function of Id genes in osteoclasts, hematopoietic cells, tumorigenesis, and metastasis is clearly necessary in order to intelligently design tailored therapies. As the molecular and cellular events that regulate the function of the BM microenvironment are unraveled, novel methods for its specific manipulation for tailored therapies should also be revealed.

## Materials and Methods

### Mice

Generation of *Id1^−/−^* mice has been previously reported [43]. Animals used in all experiments were matched for sex, age (6–8 weeks old) and genetic background (C57B6/Sv129). All animal procedures were approved and performed under the guidelines of the Institutional Animal Care and Use Committee (IACUC).

### Micro-Computed Tomography (Micro-CT)

To assess bone microarchitecture, the right femurs of the mice were removed and cleaned of all soft tissue. The bones were scanned in saline, using the Enhanced Vision Systems Model MS-8 *In Vitro* Micro-CT Scanner (GE Healthcare). 2-D projections of 4 femurs per scan, 4 hours per scan, were collected by Evolver software (GE Healthcare). Microview (GE Healthcare) was used to calculate 2-D images, 3-D volume generation, and threshold analysis.

### Mechanical Testing

To assess bone strength, the left femurs of the mice were removed and cleaned of all soft tissue. A 3-point bending test was performed by placing the bones, anterior face up, on two supports equidistant from the ends and 7 mm apart. The load was applied to the center of the femoral shaft with a velocity of 0.05 mm/second until fracture. Load displacement curves for each individual femur were recorded and used to calculate the bending moment (PL/4) and bending rigidity (mL3/48), where P is the applied load, L is the span of the support points (7 mm), and m is the slope of the linear portion of the load-displacement curve.

### Osteoclast Formation and Resorption Assays

BM cells from long bones were cultured in α–minimal essential medium (α-MEM) containing 10% fetal bovine serum (FBS), penicillin, streptomycin, and 5 ng/mL M-CSF (PeproTech) for 16 hours. Nonadherent cells were harvested and cultured for 3 more days in the presence of 30 ng/mL M-CSF. Floating cells were removed and adherent cells were used as osteoclast precursors. The cells were further cultured in medium supplemented with 30 ng/mL M-CSF and 50 ng/mL RANKL (PeproTech) for 3 days. The culture plate was stained for TRAP-positive multinuclear cells (TRAP^+^ MNCs) using the leukocyte acid phosphatase (TRAP) kit (Sigma-Aldrich). TRAP^+^ MNCs containing more than 3 nuclei were counted. For resorption assays, cells were plated on BD BioCoat Osteologic slides (BD Bioscience). After 10 days in culture in medium supplemented with 30 ng/mL M-CSF and 50 ng/mL RANKL, the cells were removed with bleach and resorption pits were visualized by von Kossa staining.

### Histology, Immunohistochemistry, and TRAP Staining

The femurs were cleaned of muscle and fixed in 4% paraformaldehyde for 48 hours. Following an overnight running water rinse, the bones were decalcified in 10% EDTA until the bones were soft and flexible, and processed in a VIP tissue processor to paraffin. Embedded samples were sectioned to a thickness of 5 µm and stained for H&E for standard histology. The following antibodies were used for immunohistochemical analysis: Procollagen type I clone SP1.D8 (Developmental Studies Hybridoma Bank, University of Iowa) and Cathepsin K clone C-16 (Santa Cruz Biotechnology). For TRAP staining, the use of hexazonium pararosanaline for localization of acid phosphatase activity was previously described [44]. When this acid phosphatase method is used histochemically, in the presence of tartrate, the resulting stain is due to TRAP activity [45]. To prevent bias, all slides were coded and a total of 10 randomly chosen fields were assessed for each slide. For determination of common bone parameters, such as quantitation of cell types, determination of bone surfaces, and mineral apposition rate, the slides were evaluated with use of the Bioquant Morphometric System (Bioquant Image Analysis Corp., Nashville, TN).

### Bone Histomorphometry

At 5 weeks of age, each animal was labeled by intraperitoneal injection with Tetracycline (5 mg/kg body weight), followed by Xlenol Orange (90 mg/kg body weight) with a 4 day interval between labels. One day after the second label, the animals were sacrificed and the femurs were removed. The bones were subsequently fixed in 10% neutral buffered formalin for forty-eight hours, washed overnight in running water, dehydrated through a graded ethanol treatment, cleared in xylene, embedded in methyl methacrylate, and sectioned to a thickness of 5 µm, as previously described [46]. The distance between the two labels was measured in 10 randomly chosen fields per slide, and the mineral apposition rate (µm/day) was determined by dividing the mean distance between the double labels by the interlabel time (4 days).

### Cell Preparation and Flow Cytometry

BM cell numbers were determined with a FACSCalibur (BD). Red blood cells were lysed using ACK buffer (Gibco, Invitrogen) according to the manufacturer's instructions. For the preparation of cells for flow cytometry, before cells were stained with specific antibodies, nonspecific binding sites were blocked, when needed, with purified anti-FcgRII/III (93; ebioscience). All cells were stained at 4°C in PBS with 5% (vol/vol) FCS. The following antibodies were used for staining: anti-CD3 (2C11), anti-CD11c (HL3), anti-CD43 (S7; all from Pharmingen); anti-CD34 (Ram34), anti-CD19 (1D3), anti-NK1.1 (PK136), anti-Ter119 (Ter119), anti-Gr1 (RB6-8C5), anti-CD11b (M1/70), anti-CD16/32 (93), anti-CD117 (2B8), anti-Sca-1 (D7), anti-CD45R (RA3-6B2), anti-IgM (11/41) and anti-CD25 (PC61.5; all from eBioscience). The following reagents were used for secondary steps: DAPI (4,6-diamino-2-phenylindole; Molecular Probes) and streptavidin-phycoerythrin-carbocyanine 5.5 (Caltag).

### Differential Blood Cell Counts

Blood samples were obtained by retro-orbital bleeding and diluted 1:4 in PBS with 2 mM EDTA, 5% BSA. Complete blood cell counts were analyzed on an ADVIA 120 (Bayer).

### Cell Cycle Analysis of Progenitor Cells

For BrdU incorporation, mice were first injected intraperitoneally with BrdU (1 mg per 6 g of mouse weight), followed by administration of BrdU in the drinking water (0.8 mg/ml) for 5 days. BM cells were harvested and stained for surface markers. Intracellular staining with anti-BrdU (PE) was carried out using the BrdU Flow Kit (BD Pharmingen) following the manufacturer's instructions. Sca-1 and c-kit antibodies were used as stem cell surface markers. For assessing *in vitro* proliferation, Lin^−^, Sca-1^+^, c-kit^+^ (LSK) stem cells were sorted from the BM into a 48 well plate (100 cells/well), and clonal expansion was followed for 4 days by manual cell counting under the microscope. Cells were cultured in StemSpan Serum Free Expansion Medium (STEMCELL Technologies) containing StemSpan CC100 Cytokine Cocktail (100 ng/ml SCF, 100 ng/ml Flt-3, 20 ng/ml IL-3, 20 ng/ml IL-6) (STEMCELL Technologies).

### Quantitative PCR (qPCR)

Femurs were frozen in liquid nitrogen and crushed with a mortar and pestle. RNA from the crushed bone was extracted using the RNAqueous kit (Ambion), and reverse transcribed using Superscript III reverse transcriptase (Invitrogen). QPCR was performed on a 7500 Fast Real Time PCR System (Applied Biosystems), using TaqMan Universal PCR Master Mix (Applied Biosystems). Relative expression was normalized to β-actin levels.

### SCF ELISA

Plasma levels of SCF were determined using the Mouse SCF Quantikine ELISA kit (R&D systems) according to manufacturer's instructions.

### 5-FU Challenge

Mice were injected intraperitoneally with 150 mg/kg 5-FU in PBS once a week.

### Bone Marrow Transplantation

Recipient mice were lethally irradiated with a single dose of 9.5 Gy of whole-body irradiation. 24 hours after irradiation, 2×10^6^ donor lineage depleted BM cells were injected by tail vein. The BM of donor mice was harvested by flushing femurs and tibias with PBS+2% FBS. Lineage negative cells were purified by lineage-marker negative selection using the Mouse Hematopoietic Progenitor Enrichment kit (StemCell Technologies). All recipient mice were female and all donor mice were male.

### Plasmids

PGEW-empty and PGEW-Id1 vectors were built from plasmid pCCL.sin.cPPT.PGK.GFP.WPRE [47]. A linker was inserted downstream of GFP for the purpose of introducing restriction sites for the insertion of the EF1α promoter and Id1. The EF1α promoter fragment (−1208 to −24) was inserted into the PshAI site of pCCL.sin.cPPT.PGK.GFP.linker.WPRE to generate the pCCL.sin.cPPT.PGK.GFP.EF1α.linker.WPRE vector, which we have termed PGEW-empty for simplicity. A 585-bp fragment containing the complete coding sequence of Id1 was obtained by PCR, using cDNA from mouse BM as a template, with oligonucleotide primers (5′-CGGAATTCCTCCGCCTGTTCTCAGGA-3′ and 5′-CAAGAAGCTTGCGGTAGTGTCTTTCC-3′). The fragment was digested with EcoRI, blunted with T4 DNA Polymerase, and lastly, digested with SalI. This fragment was then inserted into the PmeI/SalI sites of pCCL.sin.cPPT.PGK.GFP.EF1α.linker.WPRE to generate the pCCL.sin.cPPT.PGK.GFP.EF1α.ID1.WPRE vector, which we have termed PGEW-Id1 for simplicity. shCTSK (pLKO.1-puro short hairpin Ctsk, TRCN0000054626) and control shGFP were purchased (Sigma-Aldrich).

### Vector Production and Titration

Lentiviral vector stocks, pseudotyped with the vesicular stomatitis G protein (VSV-G), were produced by transient cotransfection of 293T cells and titered on HeLa cells as described [47]. Viral supernatants were concentrated to titers ≥10^8^ transduction units/ml by ultracentrifugation.

### Isolation and Transduction of Hematopoietic Cells

BM of donor mice was harvested by flushing femurs and tibias with PBS+2% FBS. Lin^−^ cells were purified by lineage-marker negative selection using the Mouse Hematopoietic Progenitor Enrichment kit (StemCell Technologies), plated at a density of 1×10^6^ cells/ml in StemSpan Serum Free Expansion Medium (STEMCELL Technologies). Lin^−^ cells were transduced with concentrated virus for 12 h (MOI of 50–60), washed, and resuspended in PBS for transplantation.

### Statistical Analysis

Statistical and graphical analyses were performed using GraphPad Prism software (version 3.0). The data was analyzed using Student's unpaired t-test and results were considered significant at the 95% significance level (P<0.05). Results were representative of two or more independent experiments, and data was expressed as mean ± SEM of at least 3 replicates.

## Supporting Information

Figure S1 *Id1^−/−^* mice weigh less and have less trabecular bone. (A) Weights of 6-week old wild-type and *Id1^−/−^* mice (***P<0.001; n = 12). Error bars represent±S.E.M. (B) Representative H&E staining of femoral sections from wild-type and *Id1^−/−^* mice. Arrowheads indicate areas of trabecular bone; M, marrow; GP, growth plate.
(2.17 MB PPT)

Figure S2 Absence of Id1 does not alter the expression of osteoblast-associated genes. Results of qPCR for the expression of RANKL, OPG, and SDF-1 in the BM of wild-type and *Id1^−/−^* mice (n = 6). Error bars represent ±S.E.M.
(0.04 MB PPT)

Figure S3 Detection of Y chromosome DNA sequences in BM transplanted mice. DNA samples were isolated from 200 µL of peripheral blood from lethally irradiated wild-type and *Id1^−/−^* mice that were transplanted with either wild-type or *Id1^−/−^* BM. Control DNA was isolated from wild-type male and female mice, and admixed to generate standards with known ratios of male and female DNA. Thus, XY male DNA was serially diluted in XX female DNA. Standards and samples were assayed by using TaqMan Gene Expression Assays (Applied Biosystems) for the sex determining region (SRY) gene. The cycle threshold (Ct) readings of the standards were used to generate a standard curve by plotting the mean of triplicate Ct values versus the log of the percentage of Y DNA in the background of XX DNA and calculating a regression line. The amount of Y DNA in unknown samples was determined by applying the mean Ct value of triplicates to the standard curve and correcting for the total amount of DNA in the sample to determine the percentage of male sequence within a female background. Error bars represent ±S.E.M.
(0.04 MB PPT)

Figure S4 Absence of Id1 specifically upregulates the expression of CTSK and not other cathepsins. Results of qPCR for the expression of other cathepsin family genes, CTSL and CTSB in the BM of wild-type and *Id1^−/−^* mice (n = 6). Error bars represent ±S.E.M.
(0.04 MB PPT)

Figure S5 A model for the role of Id1 in regulating myeloid and osteoclast differentiation. Id1 inhibition of myeloid and osteoclast differentiation regulates HSC niche factors and limits HSC mobilization (left). In the absence of Id1, osteoclast differentiation increases and results in increased CTSK secretion (right).
(0.07 MB PPT)

Figure S6 Use of lentiviral vectors for the overexpression of Id1. (A) Schematic drawings of the lentiviral vector containing Id1 (PGEW-Id1) and the empty vector control (PGEW-empty). Both vectors contain the promoter of the elongation factor 1 alpha (EF1α) gene and carry an internal cassette for the enhanced green fluorescent protein (EGFP) driven by the promoter of the human phosphoglycerate kinase (PGK) gene. The following viral cis-acting sequences are labeled: long terminal regions (LTR); major splice donor sites (SD), encapsidation signal (ψ) including the 5′ portion of the gag gene (GA); Rev-response element (RRE); splice acceptor sites (SA); and post-transcriptional regulatory element of woodchuck hepatitis virus (Wpre). (B) Expression of Id1 in the BM of transplanted mice (***P<0.001; n = 6). Lin- BM cells from *Id1^−/−^* mice were transduced with lentivirus containing PGEW-Id1 or PGEW-empty vector overnight and transplanted into lethally irradiated *Id1^−/−^* mice. After 8 weeks, the mice were sacrificed and BM from the femur was collected for qPCR analysis. Error bars represent ±S.E.M.
(0.05 MB PPT)

Figure S7 Use of lentiviral vectors to knockdown expression of CTSK. (A) Expression of CTSK in the BM of transplanted mice (***P<0.001; n = 6). Lin- BM cells from *Id1^−/−^* mice were transduced with lentivirus containing shCTSK or shGFP vector overnight and transplanted into lethally irradiated *Id1^−/−^* mice. After 3.5 months, the mice were sacrificed and BM from the femur was collected for qPCR analysis. Error bars represent ±S.E.M. (B) Representative H&E staining of femoral sections from *Id1^−/−^* mice transplanted with BM containing a shRNA targeted against CTSK or GFP. Arrowheads indicate areas of trabecular bone; M, marrow; GP, growth plate.
(1.85 MB PPT)

Table S1 Steady state peripheral blood cell counts in wild-type and *Id1^−/−^* mice.
(0.06 MB PPT)

Table S2 Characteristics of femurs in CTSK-shRNA and GFP-shRNA BM transplanted mice.
(0.05 MB PPT)
