# Supplementary figures and images for: Id1 Represses Osteoclast-Dependent Transcription and Affects Bone Formation and Hematopoiesis
Source: PLoS One. 2009 Nov 24;4(11):e7955. doi: 10.1371/journal.pone.0007955 (PMC2776978; doi:10.1371/journal.pone.0007955)

## Slide 1
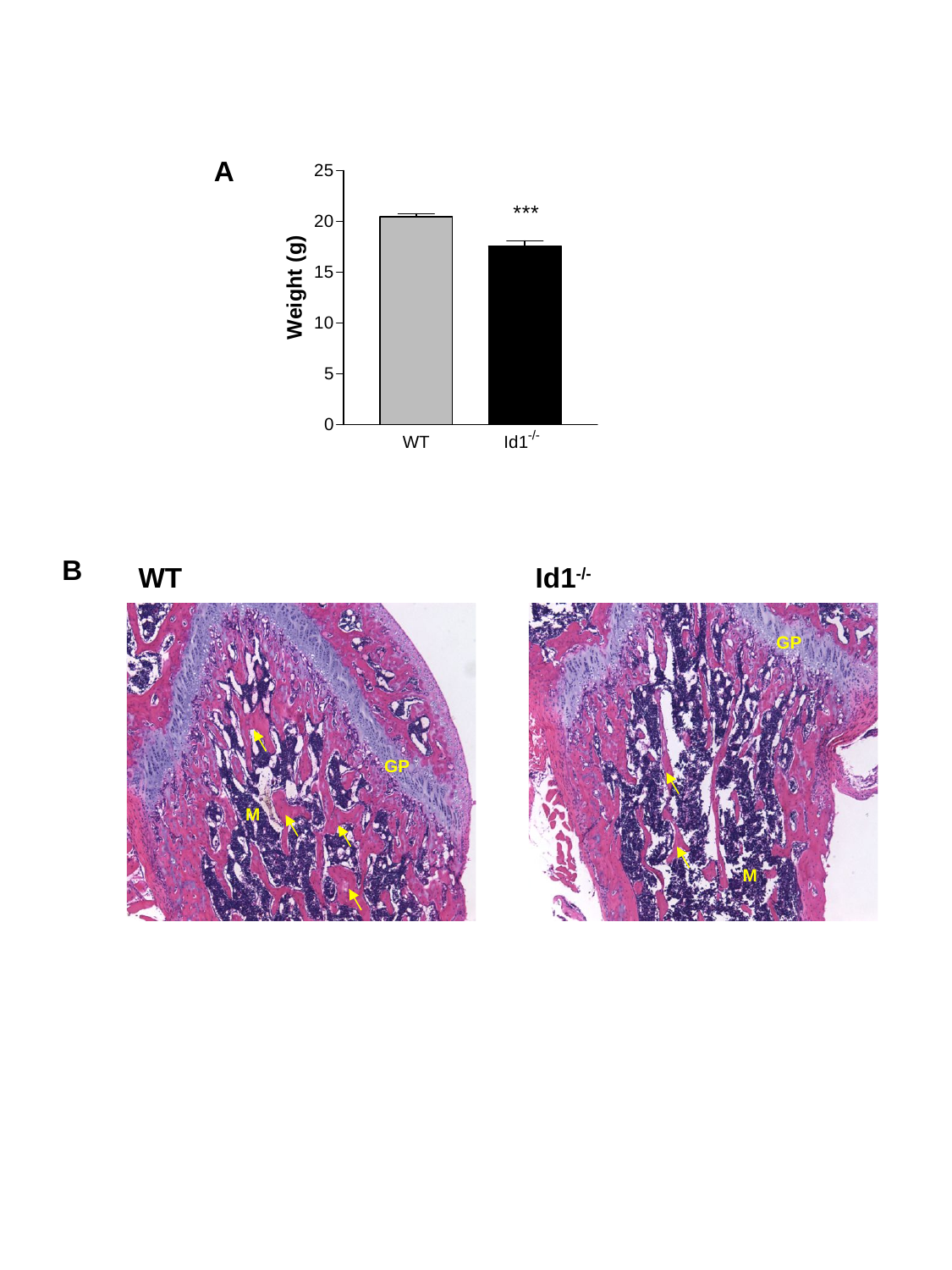

A
B
WT
Id1-/-
GP
GP
M
M

Supplement: Figure S1 — Id1−/− mice weigh less and have less trabecular bone. (A) Weights of 6-week old wild-type and Id1−/− mice (***P<0.001; n = 12). Error bars represent±S.E.M. (B) Representative H&E staining of femoral sections from wild-type and Id1−/− mice. Arrowheads indicate areas of trabecular bone; M, marrow; GP, growth plate. (2.17 MB PPT) [file pone.0007955.s001.ppt]

## Slide 1
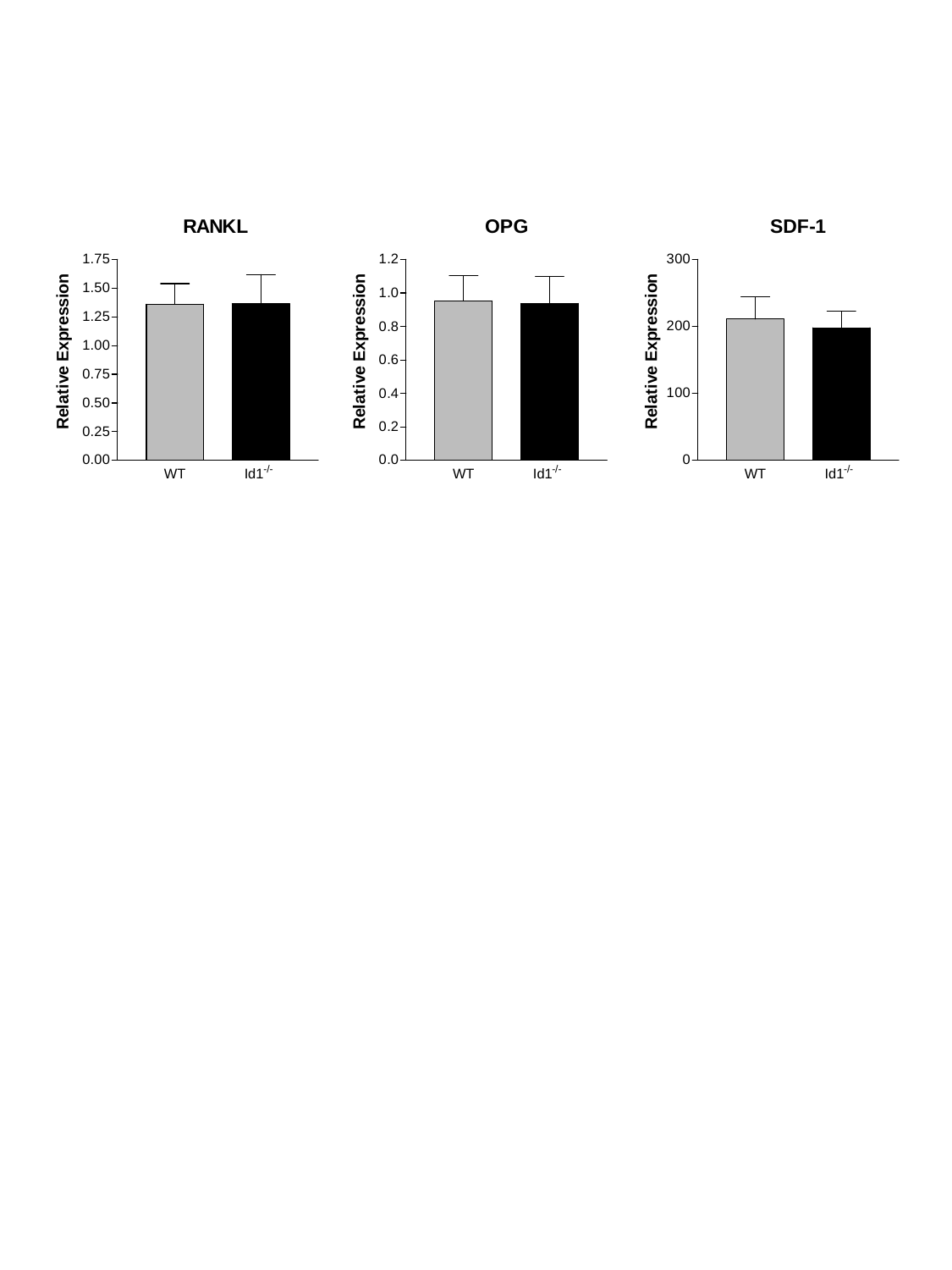

Supplement: Figure S2 — Absence of Id1 does not alter the expression of osteoblast-associated genes. Results of qPCR for the expression of RANKL, OPG, and SDF-1 in the BM of wild-type and Id1−/− mice (n = 6). Error bars represent ±S.E.M. (0.04 MB PPT) [file pone.0007955.s002.ppt]

## Slide 1
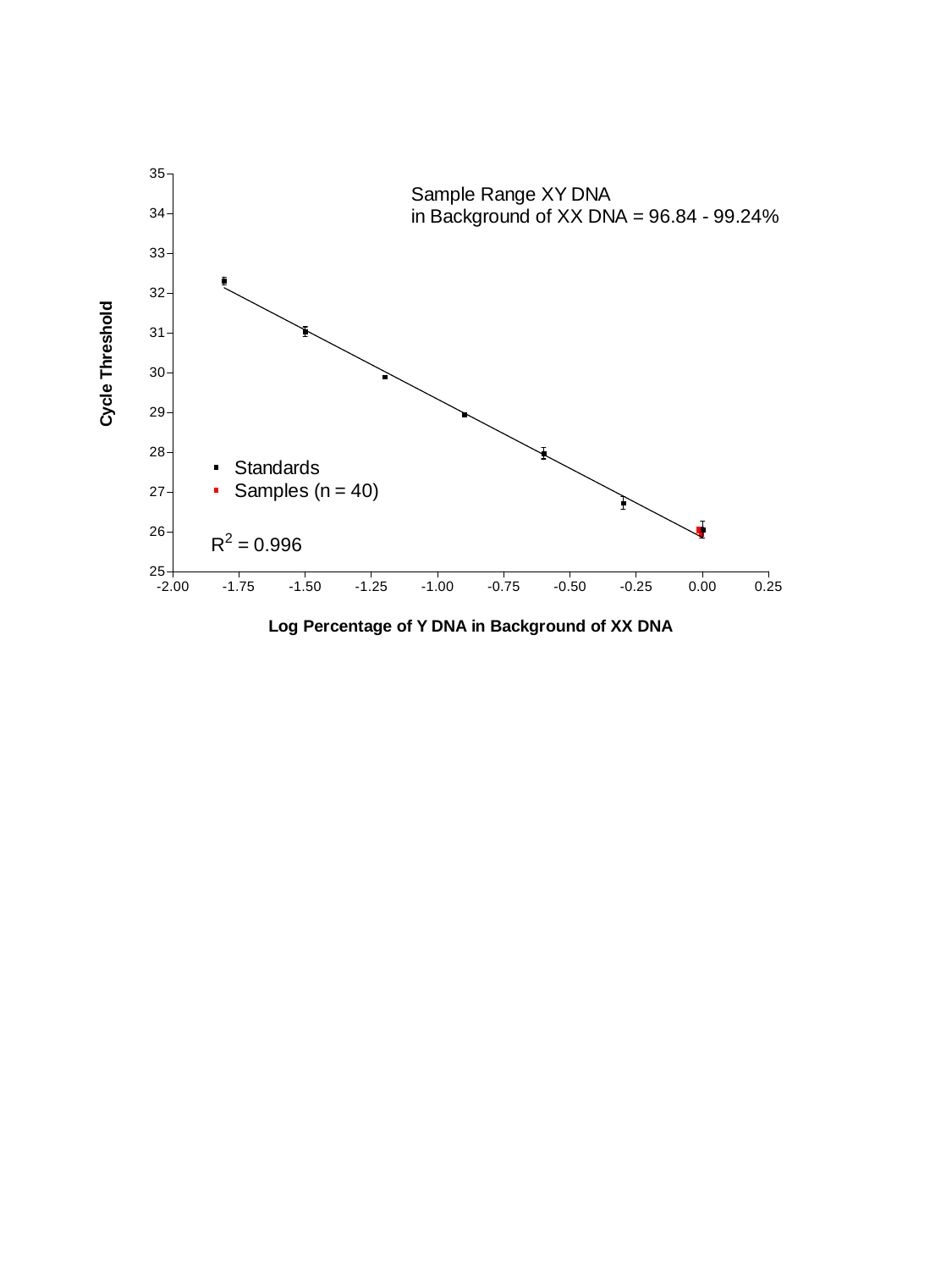

Supplement: Figure S3 — Detection of Y chromosome DNA sequences in BM transplanted mice. DNA samples were isolated from 200 µL of peripheral blood from lethally irradiated wild-type and Id1−/− mice that were transplanted with either wild-type or Id1−/− BM. Control DNA was isolated from wild-type male and female mice, and admixed to generate standards with known ratios of male and female DNA. Thus, XY male DNA was serially diluted in XX female DNA. Standards and samples were assayed by using TaqMan Gene Expression Assays (Applied Biosystems) for the sex determining region (SRY) gene. The cycle threshold (Ct) readings of the standards were used to generate a standard curve by plotting the mean of triplicate Ct values versus the log of the percentage of Y DNA in the background of XX DNA and calculating a regression line. The amount of Y DNA in unknown samples was determined by applying the mean Ct value of triplicates to the standard curve and correcting for the total amount of DNA in the sample to determine the percentage of male sequence within a female background. Error bars represent ±S.E.M. (0.04 MB PPT) [file pone.0007955.s003.ppt]

## Slide 1
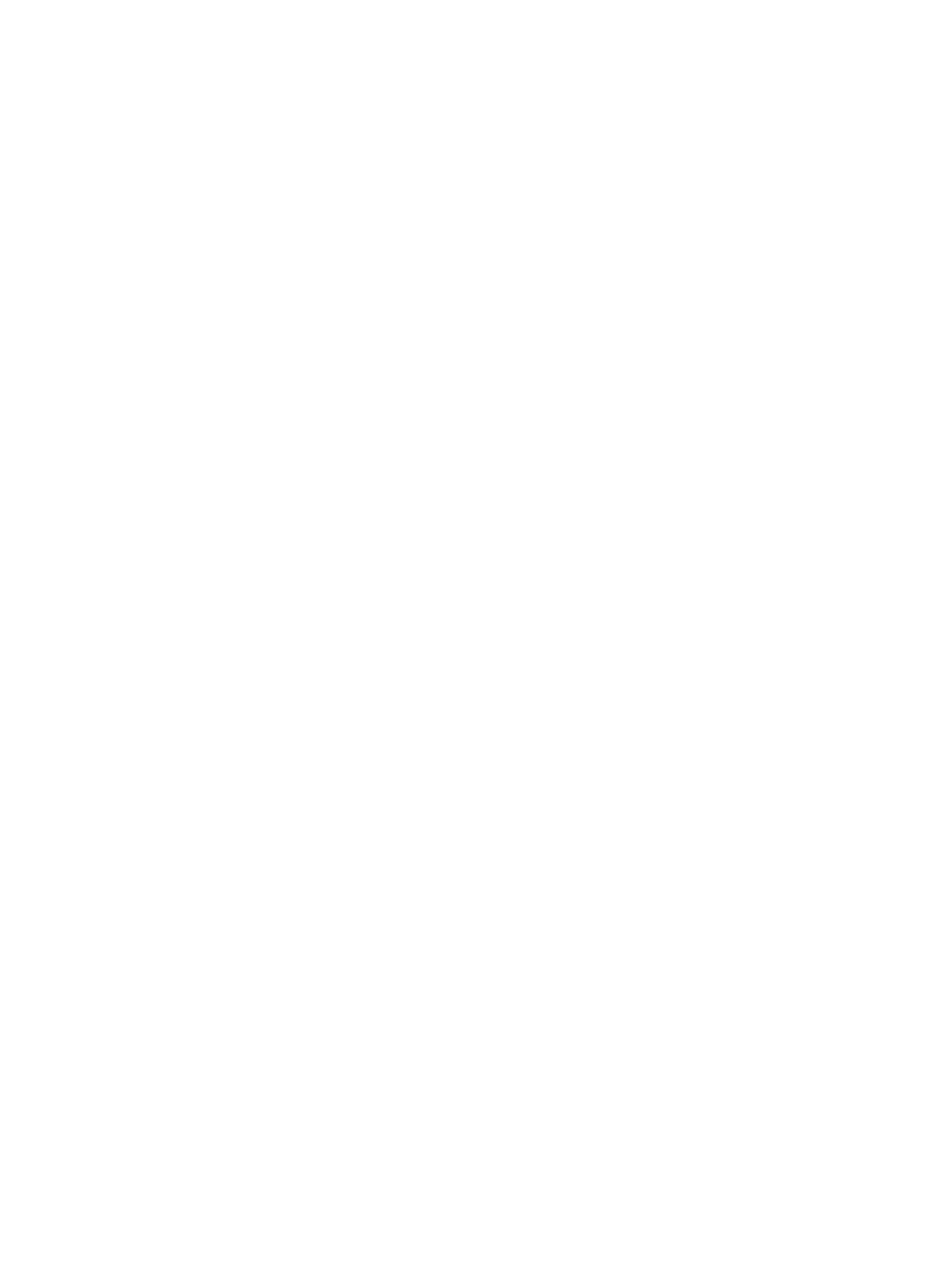

Supplement: Figure S4 — Absence of Id1 specifically upregulates the expression of CTSK and not other cathepsins. Results of qPCR for the expression of other cathepsin family genes, CTSL and CTSB in the BM of wild-type and Id1−/− mice (n = 6). Error bars represent ±S.E.M. (0.04 MB PPT) [file pone.0007955.s004.ppt]

## Slide 1
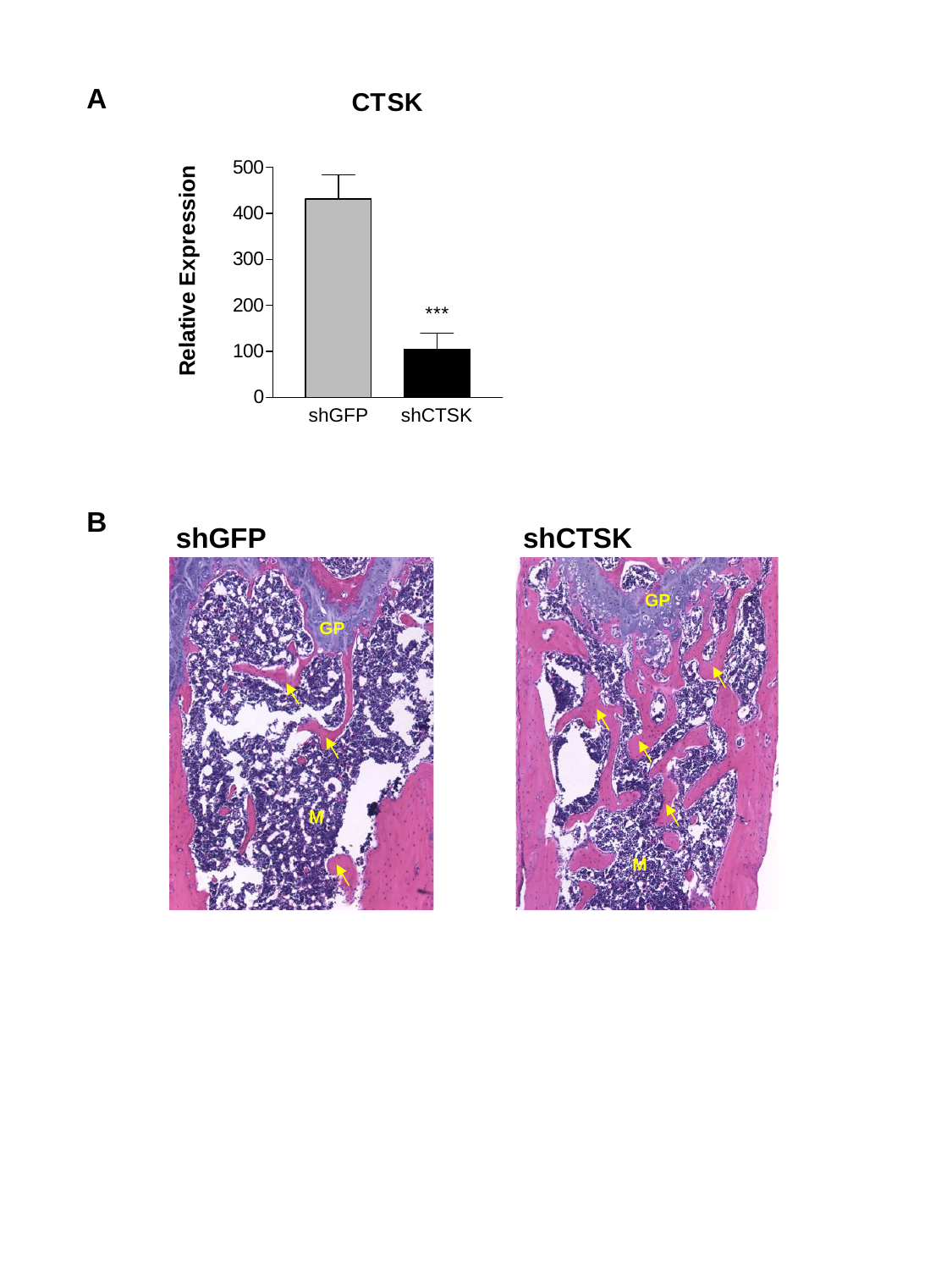

A
B
shGFP
shCTSK
GP
M
GP
M

Supplement: Figure S7 — Use of lentiviral vectors to knockdown expression of CTSK. (A) Expression of CTSK in the BM of transplanted mice (***P<0.001; n = 6). Lin- BM cells from Id1−/− mice were transduced with lentivirus containing shCTSK or shGFP vector overnight and transplanted into lethally irradiated Id1−/− mice. After 3.5 months, the mice were sacrificed and BM from the femur was collected for qPCR analysis. Error bars represent ±S.E.M. (B) Representative H&E staining of femoral sections from Id1−/− mice transplanted with BM containing a shRNA targeted against CTSK or GFP. Arrowheads indicate areas of trabecular bone; M, marrow; GP, growth plate. (1.85 MB PPT) [file pone.0007955.s007.ppt]

## Slide 1
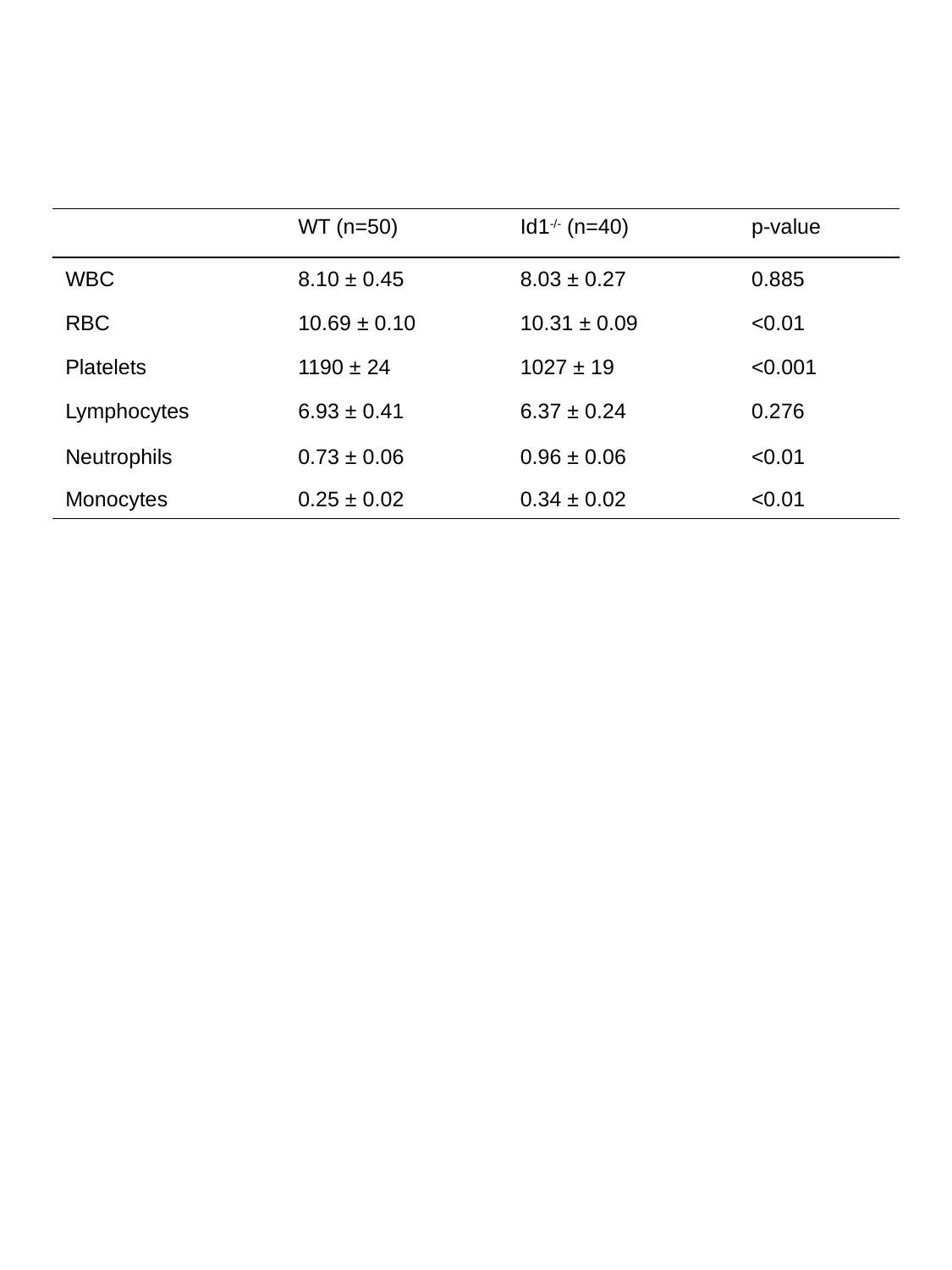

| | WT (n=50) | Id1-/- (n=40) | p-value |
| --- | --- | --- | --- |
| WBC | 8.10 ± 0.45 | 8.03 ± 0.27 | 0.885 |
| RBC | 10.69 ± 0.10 | 10.31 ± 0.09 | <0.01 |
| Platelets | 1190 ± 24 | 1027 ± 19 | <0.001 |
| Lymphocytes | 6.93 ± 0.41 | 6.37 ± 0.24 | 0.276 |
| Neutrophils | 0.73 ± 0.06 | 0.96 ± 0.06 | <0.01 |
| Monocytes | 0.25 ± 0.02 | 0.34 ± 0.02 | <0.01 |

Supplement: Table S1 — Steady state peripheral blood cell counts in wild-type and Id1−/− mice. (0.06 MB PPT) [file pone.0007955.s008.ppt]
