# Supplementary material for: Id1 Represses Osteoclast-Dependent Transcription and Affects Bone Formation and Hematopoiesis
Source: PLoS One. 2009 Nov 24;4(11):e7955. doi: 10.1371/journal.pone.0007955 (PMC2776978; doi:10.1371/journal.pone.0007955)
Supplement: Figure S5 — A model for the role of Id1 in regulating myeloid and osteoclast differentiation. Id1 inhibition of myeloid and osteoclast differentiation regulates HSC niche factors and limits HSC mobilization (left). In the absence of Id1, osteoclast differentiation increases and results in increased CTSK secretion (right). (0.07 MB PPT) [file pone.0007955.s005.ppt]

## Slide 1
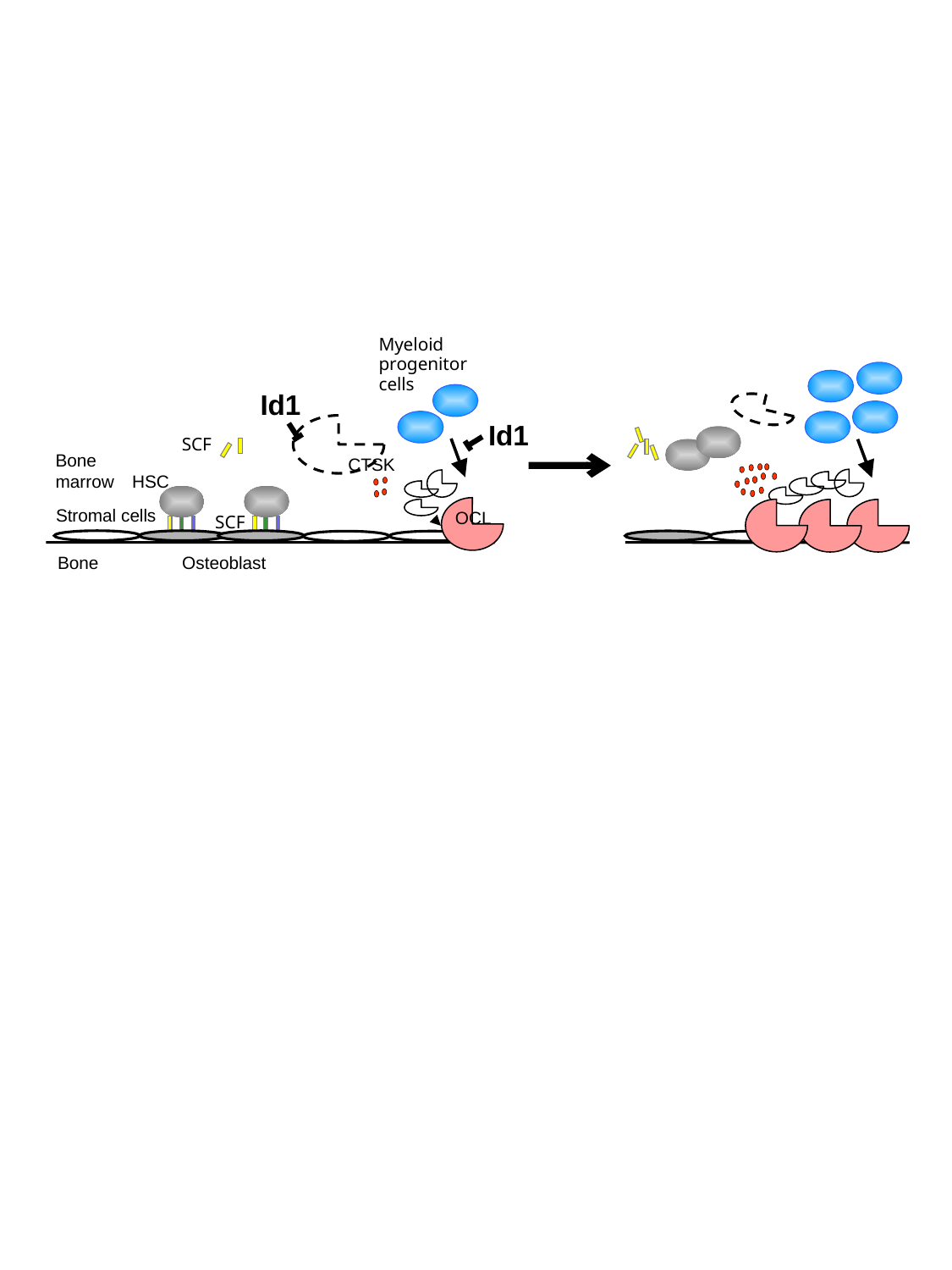

Myeloid progenitor cells
Id1
Id1
SCF
Bone
marrow
CTSK
HSC
Stromal cells
OCL
SCF
Osteoblast
Bone
