# Supplementary material for: Id1 Represses Osteoclast-Dependent Transcription and Affects Bone Formation and Hematopoiesis
Source: PLoS One. 2009 Nov 24;4(11):e7955. doi: 10.1371/journal.pone.0007955 (PMC2776978; doi:10.1371/journal.pone.0007955)
Supplement: Figure S6 — Use of lentiviral vectors for the overexpression of Id1. (A) Schematic drawings of the lentiviral vector containing Id1 (PGEW-Id1) and the empty vector control (PGEW-empty). Both vectors contain the promoter of the elongation factor 1 alpha (EF1α) gene and carry an internal cassette for the enhanced green fluorescent protein (EGFP) driven by the promoter of the human phosphoglycerate kinase (PGK) gene. The following viral cis-acting sequences are labeled: long terminal regions (LTR); major splice donor sites (SD), encapsidation signal (ψ) including the 5′ portion of the gag gene (GA); Rev-response element (RRE); splice acceptor sites (SA); and post-transcriptional regulatory element of woodchuck hepatitis virus (Wpre). (B) Expression of Id1 in the BM of transplanted mice (***P<0.001; n = 6). Lin- BM cells from Id1−/− mice were transduced with lentivirus containing PGEW-Id1 or PGEW-empty vector overnight and transplanted into lethally irradiated Id1−/− mice. After 8 weeks, the mice were sacrificed and BM from the femur was collected for qPCR analysis. Error bars represent ±S.E.M. (0.05 MB PPT) [file pone.0007955.s006.ppt]

## Slide 1
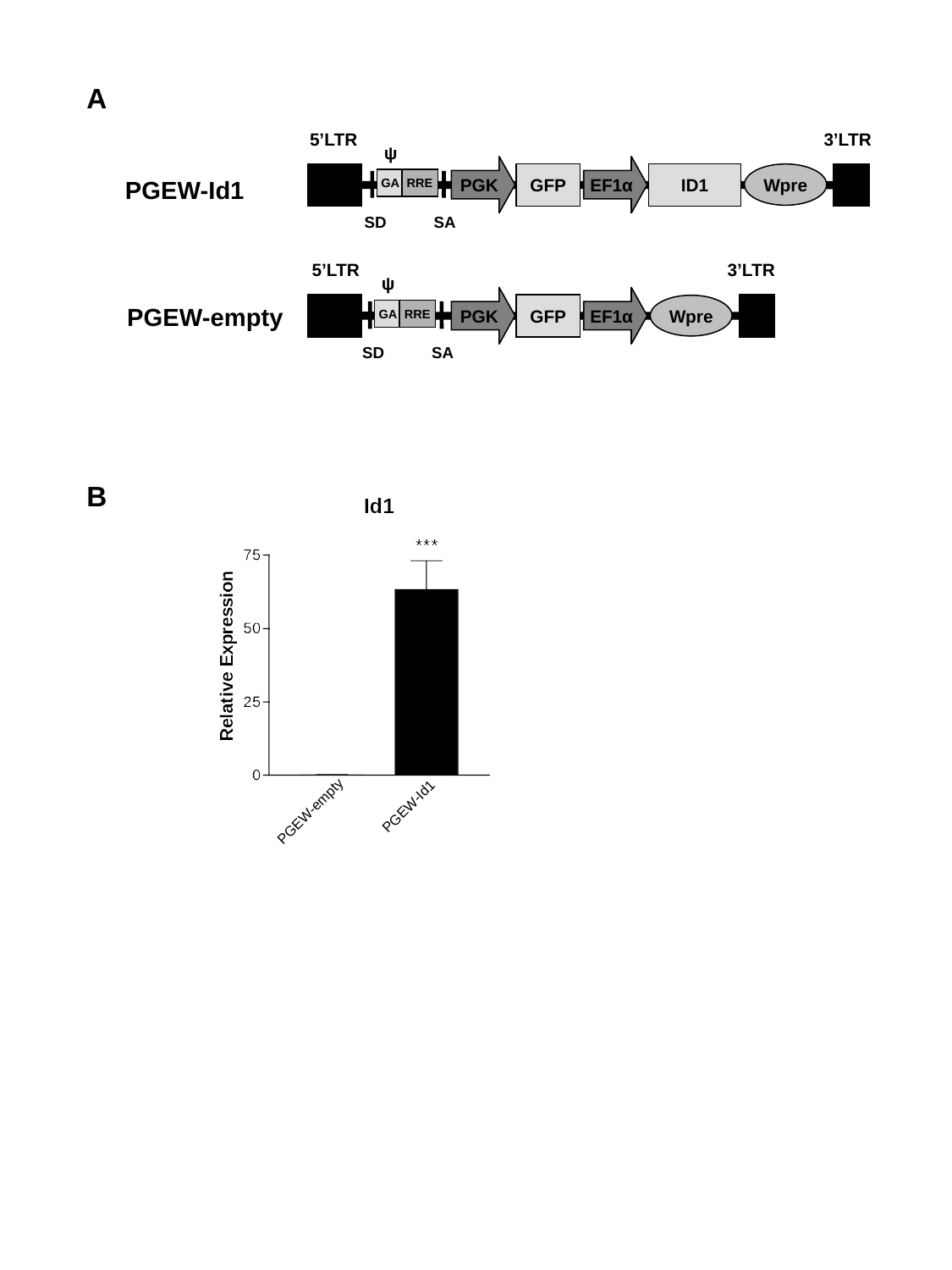

A
5’LTR
3’LTR
ψ
PGK
EF1α
GFP
ID1
Wpre
PGEW-Id1
GA
RRE
SD
SA
5’LTR
3’LTR
ψ
PGK
EF1α
GFP
Wpre
PGEW-empty
GA
RRE
SD
SA
B
PGEW-Id1
PGEW-empty
