# Supplementary material for: Id1 Represses Osteoclast-Dependent Transcription and Affects Bone Formation and Hematopoiesis
Source: PLoS One. 2009 Nov 24;4(11):e7955. doi: 10.1371/journal.pone.0007955 (PMC2776978; doi:10.1371/journal.pone.0007955)
Supplement: Table S2 — Characteristics of femurs in CTSK-shRNA and GFP-shRNA BM transplanted mice. (0.05 MB PPT) [file pone.0007955.s009.ppt]

## Slide 1
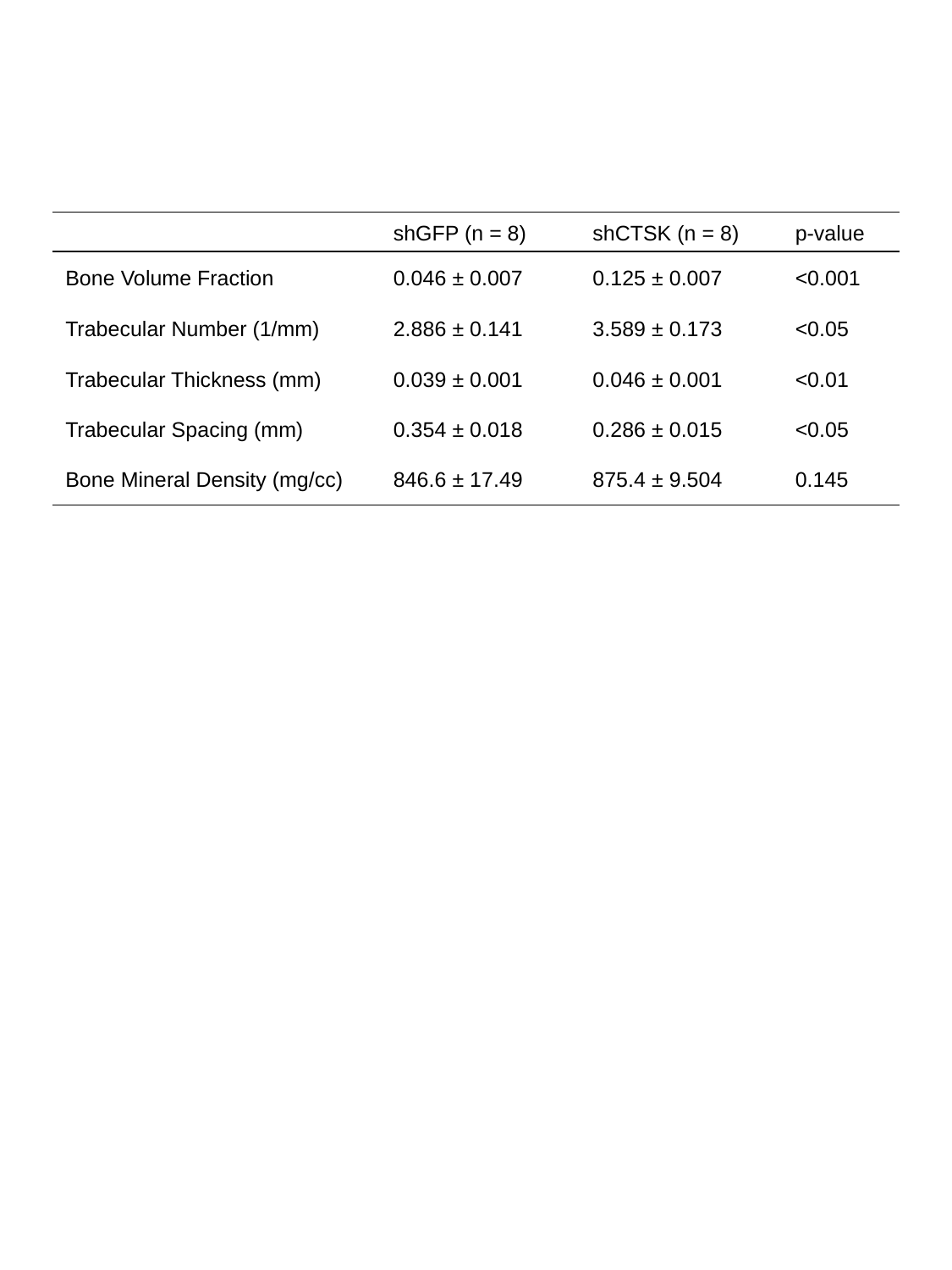

| | shGFP (n = 8) | shCTSK (n = 8) | p-value |
| --- | --- | --- | --- |
| Bone Volume Fraction | 0.046 ± 0.007 | 0.125 ± 0.007 | <0.001 |
| Trabecular Number (1/mm) | 2.886 ± 0.141 | 3.589 ± 0.173 | <0.05 |
| Trabecular Thickness (mm) | 0.039 ± 0.001 | 0.046 ± 0.001 | <0.01 |
| Trabecular Spacing (mm) | 0.354 ± 0.018 | 0.286 ± 0.015 | <0.05 |
| Bone Mineral Density (mg/cc) | 846.6 ± 17.49 | 875.4 ± 9.504 | 0.145 |
